# Clinico-Pathological and Clinical Outcomes of Soft Tissue Sarcoma With FUS-TFCP2 or EWSR1-TFCP2 Fusions: A Case Series From North India

**DOI:** 10.7759/cureus.98552

**Published:** 2025-12-05

**Authors:** Karishma Agarwal, Sameer Rastogi, Sunil Pasricha, Yamini Dharmashaktu, Aditi Aggarwal

**Affiliations:** 1 Radiation Oncology, Max Super Speciality Hospital, Saket, New Delhi, IND; 2 Oncology, All India Institute of Medical Sciences, New Delhi, New Delhi, IND; 3 Pathology, All India Institute of Medical Sciences, New Delhi, New Delhi, IND; 4 Nuclear Medicine, All India Institute of Medical Sciences, New Delhi, New Delhi, IND

**Keywords:** ewsr1 mutation, fus-tfcp2 mutation, ihc panel, ngs panel, radiotherapy, rhabdomyosarcoma, sarcoma, soft tissue sarcoma, tfcp2 mutation, treatment of sarcoma

## Abstract

Tumours with FUS-TFCP2 or EWSR1-TFCP2 mutations are classified under rhabdomyosarcomas at present. These tumours are exceedingly rare and on pathology show spindled to epitheloid cells, with a predisposition for gnathic bone involvement, with an aggressive outcome.

We hereby present a total of four patients harbouring a FUS-EWSR1 TFCP2 mutation recruited over a period of 2.5 years. All four reported cases are males with median age of 26.5 years (range 22-37 years). Primary site in all four cases was bone, with three being of head and neck origin and one with a primary in femur. Three of our patients were misdiagnosed outside.

All of our patients received chemotherapy at some point and three out of four cases received radiation with a good response (two patients with palliative doses of radiation also responded well). All four patients are alive at follow-up, two on treatment for metastatic disease. This is the first Indian case series reporting the clinicopathological details in patients with TFCP2 mutations.

## Introduction

Soft tissue sarcomas account for less than 1% of adult head and neck malignancies, of which 2-5% represent rhabdomyosarcomas (RMS) [1]. Rhabdomyosarcomas of the head and neck are encountered more frequently in children and adolescents with adults being affected in less than 10% cases [2].

Major histological subtypes of RMS are embryonal (ERMS), alveolar (ARMS), pleomorphic (PRMS), and spindle cell/sclerosing rhabdomyosarcoma. ERMS and ARMS are more commonly seen in the paediatric age group. ARMS are commonly seen in adolescents, typically characterized by FOXO1 gene rearrangement, and are clinically more aggressive than ERMS [3]. Spindle cell RMS was traditionally included as a variant of ERMS but it is now classified separately in the latest World Health Organization (WHO) classification (5th Edition, 2020).

Spindle cell rhabdomyosarcoma might harbour gene fusions involving VGLL2-related fusions or MYOD1-L122R mutations. Recent advances in molecular diagnostics have led to the identification of an exceedingly rare tumour currently sub-classified under RMS and carries FUS-TFCP2 or EWSR1-TFCP2 fusions. As known, EWSR1 and FUS proteins are members of the FET (FUS, EWS, TAF15) RNA binding family, this variant is also known as FET-TFCP2 RMS [4]. These tumors have a monomorphic proliferation of spindle to epithelioid cells, with a high propensity for bony involvement especially in gnathic bones, with an extremely aggressive clinical course [5]. These tumours commonly show diffuse immuno-expression of cytokeratin (CK) and anaplastic lymphoma kinase (ALK), which poses a diagnostic challenge. The treatment protocol for conventional RMS is not usually effective in these cases.

In this multi-institutional study, due to the rarity of these tumours we hereby report a case series (four cases) of head and neck RMS in adults harbouring TFCP2 mutations along with diagnostic, therapeutic and prognostic details.

## Case presentation

Methods

This was a retrospective study done over 2.5 years from February 2023 to July 2025. Ethics approval was taken as per institutional protocol. Retrospective analysis was done based on template charts. All pathologies were reviewed by expert sarcoma pathologists and all four patients had next-generation sequencing (NGS) done from the Sarcoma Panel from MedGenome (Bangalore, India).

Results

There were a total of four patients with a median age of 26.5 years (range- 22 to 37 years), harbouring a FUS-EWSR1 TFCP2 mutation during this period of 2.5 years. Salient clinical features are presented in Table 1 and pathological features are mentioned in Table 2, respectively.

**Table 1:** Clinical presentation NACT- neo-adjuvant chemotherapy, VAC- vincristine, actinomycin-D and cyclophosphamide, NED- no evidence of disease, IE- ifosfamide and etoposide

	CASE 1	CASE 2	CASE 3	CASE 4
Age (years)	24	31	22	37
Sex	Male	Male	Male	Male
Site	Mandible	Hard palate	Right femur	Right maxilla
Presenting complaints	Sensitivity in left lower alveolus	Swelling over the hard palate	Pain in the right thigh	Headache and watering from right nose and eye
Duration of symptoms	3 months	8 months	3 months	2 months
Staging	Localised	Localised	Metastatic	Localised
Mutation	FUS-TFCP2 fusion	EWSR1-TFCP2 fusion	FUS-TFCP2 fusion	EWSR1-TFCP2 fusion
Treatment received	NACT, surgery, chemoradiation, adjuvant chemotherapy	Surgery, adjuvant chemotherapy	Surgery (excisional biopsy), radiotherapy (palliative), chemotherapy	Surgery, chemotherapy, radiotherapy (palliative)
Chemotherapy/Targeted therapy	vincristine, doxorubicin and cyclophosphamide	VAC	VAC, Single agent doxorubicin f/b IE now on cabozantinib	Ifosfamide and doxorubicin, VAC, IE, gemcitabine-docetaxel, topotecan, now on cabozantinib
Current status	NED	NED	Partial response on cabozantinib	Stable disease on cabozantinib
Disease-free interval (from treatment completion)	10 months	8 months	Ongoing treatment	Ongoing treatment

**Table 2:** Histo-morphological findings IHC- immunohistochemistry, CK- cytokeratin, SMA- alpha-smooth muscle actin, ALK- anaplastic lymphoma kinase, CD34- cluster of differentiation 34, TLE-1- transducer like enhancer of split 1, SAT B2- special AT rich sequence binding protein 2

Pathology	CASE 1	CASE 2	CASE 3	CASE 4
Morphology	spindled to epithelioid cells	spindle cells	spindle cells	spindle cells
IHC MARKERS				
CK	+ve	+ve	+ve	+ve
Desmin	+ve	+ve	+ve(focal)	-ve
MyoD1	+ve	+ve	+ve	+ve
SMA	+ve	+ve	-ve	Patchy positive
ALK	+ve	+ve	+ve	+ve
P40	-ve	-ve	-ve	-ve
CD34	-ve	-ve	-ve	-ve
S100	-ve	-ve	-ve	-ve
Myogenin	-ve	+ve	+ve	-ve
SOX 10	-ve	-ve	-ve	-ve
TLE-1	-ve	-ve	-ve	-ve
SAT B2	-	-	+ve	+ve

Case summary 1

A 24-year-old male presented with a swelling in lower jaw (clinical features as in Table 1) and underwent biopsy which was suggestive of malignant spindle cell neoplasm with significant atypia and mitosis. Subsequent histo-morphological and immunohistochemistry (IHC) findings (Table 2) and NGS sarcoma panel from MedGenome were suggestive of a diagnosis of RMS with positive oncogenic fusion of FUS-TFCP2. 

**Figure 1:**
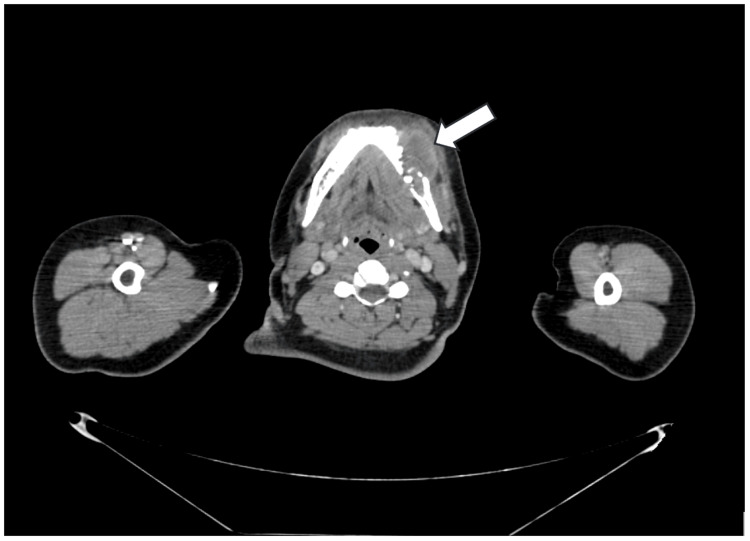
Lesion measuring 4.7*4.1*4.3 cm in left posterior buccal mucosa infiltrating the lower gingiva-buccal sulcus, alveolar process and left hemimandible.

Computed tomography (CT) scan (Figure 1) revealed a mandibular lesion with a soft tissue component, extending into gingivobuccal sulcus and left buccal mucosa, approximately 38 x 17 x 20 mm with few enlarged left level II lymph nodes largest measuring 17 x 9 mm. After this, staging workup was done and positron emission tomography/CT (PET-CT) was suggestive of locoregional disease. Fine needle aspiration cytology (FNAC) from neck nodes showed reactive lymphocytes.

He underwent three cycles of neo-adjuvant chemotherapy (NACT) with vincristine, actinomycin-D and cyclophosphamide (VAC); actinomycin-D was changed to doxorubicin due to clinical progression after one cycle. 

Post NACT imaging showed stable disease, he was then planned for surgery and he underwent wide local excision (WLE) + left extended mandibulectomy + left level I-III lymph node dissection with neck dissection with free fibula flap. Postoperative histopathology is detailed in Table 2. All resected margins and lymph nodes were free of tumor. 

He received adjuvant radiation to post op bed with margins along with Level Ia, left level Ib and II to a dose of 45 Gy in 25 fractions over five weeks along with two cycles of concurrent chemotherapy with vincristine and cyclophosphamide (q3 weekly) considering the uncommon histology with likely aggressive nature. Treatment was well tolerated with no significant toxicities.

He then received four cycles of adjuvant chemotherapy with vincristine, doxorubicin and cyclophosphamide. First follow up imaging after treatment was suggestive of no evidence of disease (NED) and he is doing well, being disease free for 10 months (till last follow up in July 2025) with a follow up of 20 months since diagnosis.

Case summary 2

A 31-year-old male, initially evaluated elsewhere, presented with widening of space between the upper incisors and swelling over the hard-palate slowly progressing in size, for which he consulted a dentist and underwent excision of the lesion along with extraction of the middle two incisors. Microscopy findings were suggestive of peripheral ossifying fibroma at an outside dental hospital.

The swelling reappeared within the next three weeks and patient was referred to a tertiary cancer care hospital, after which a re-biopsy was done. Microscopy and IHC as detailed in Table 2. On NGS sarcoma panel from MedGenome, EWSR1-TFCP2 fusion oncogenic was detected, suggestive of RMS with TFCP2 mutation, suggesting that he was initially misdiagnosed.

Patient subsequently underwent pre-maxillectomy, right inferior partial maxillectomy and bilateral nasal floor resection following which he received 10 cycles of adjuvant chemotherapy with VAC over next 10 months.

Currently, after 12 months of completion of adjuvant chemotherapy, patient is on active surveillance with NED with a follow up of 30 months since diagnosis.

Case summary 3

A 22-year-old male presented with complains of pain in right knee. An initial X-ray was suggestive of cystic lesion in right femur. MRI was suggestive of fibrous dysplasia. After which bone grafting and nailing was done and biopsy findings confirmed fibrous dysplasia, reported elsewhere. After an interval of three months, he developed pain in the right thigh. 

**Figure 2:**
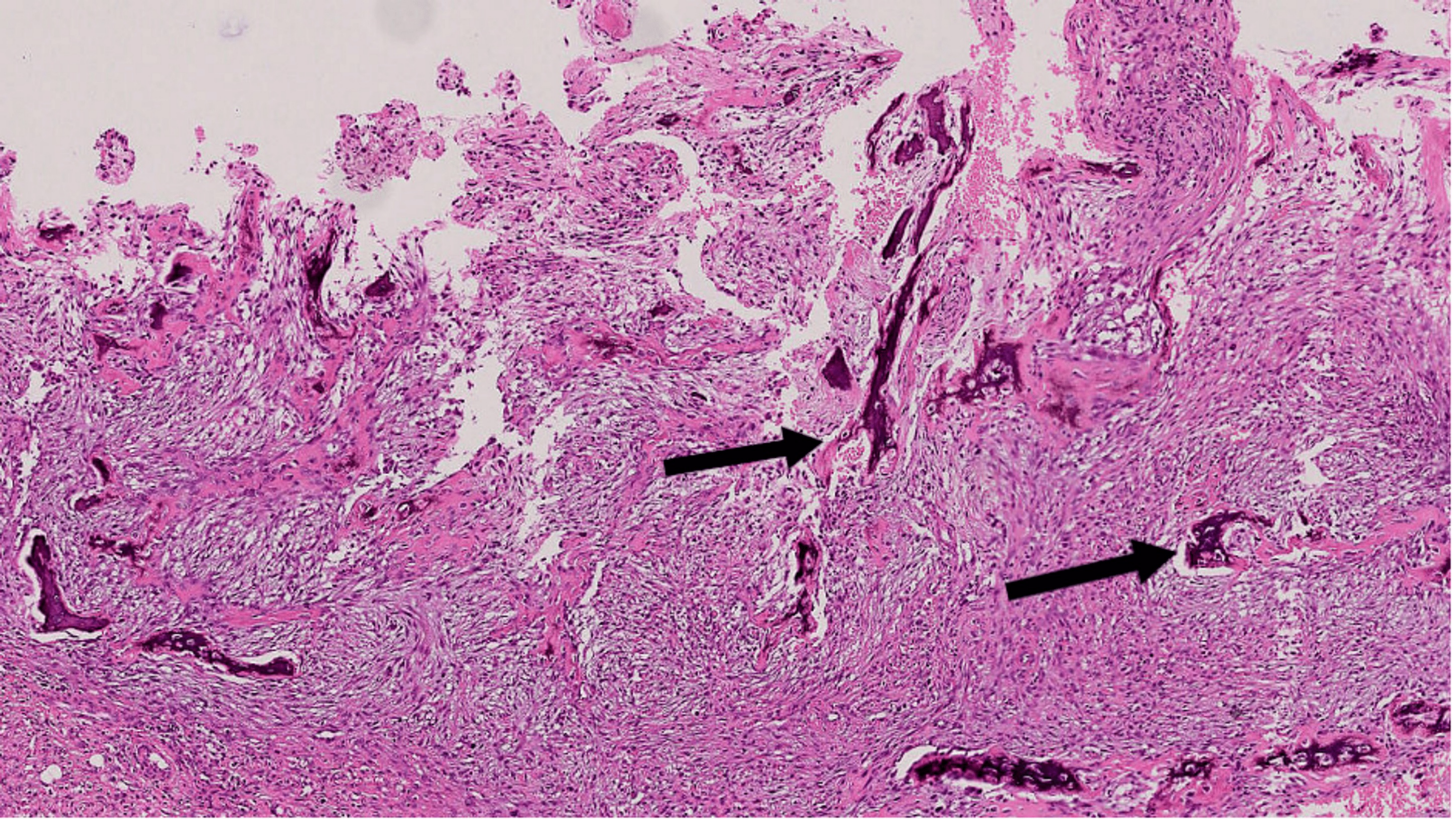
Sections show spindle cell tumor extensively infiltrating the bony trabeculae (arrow) - H&E; x40

**Figure 3:**
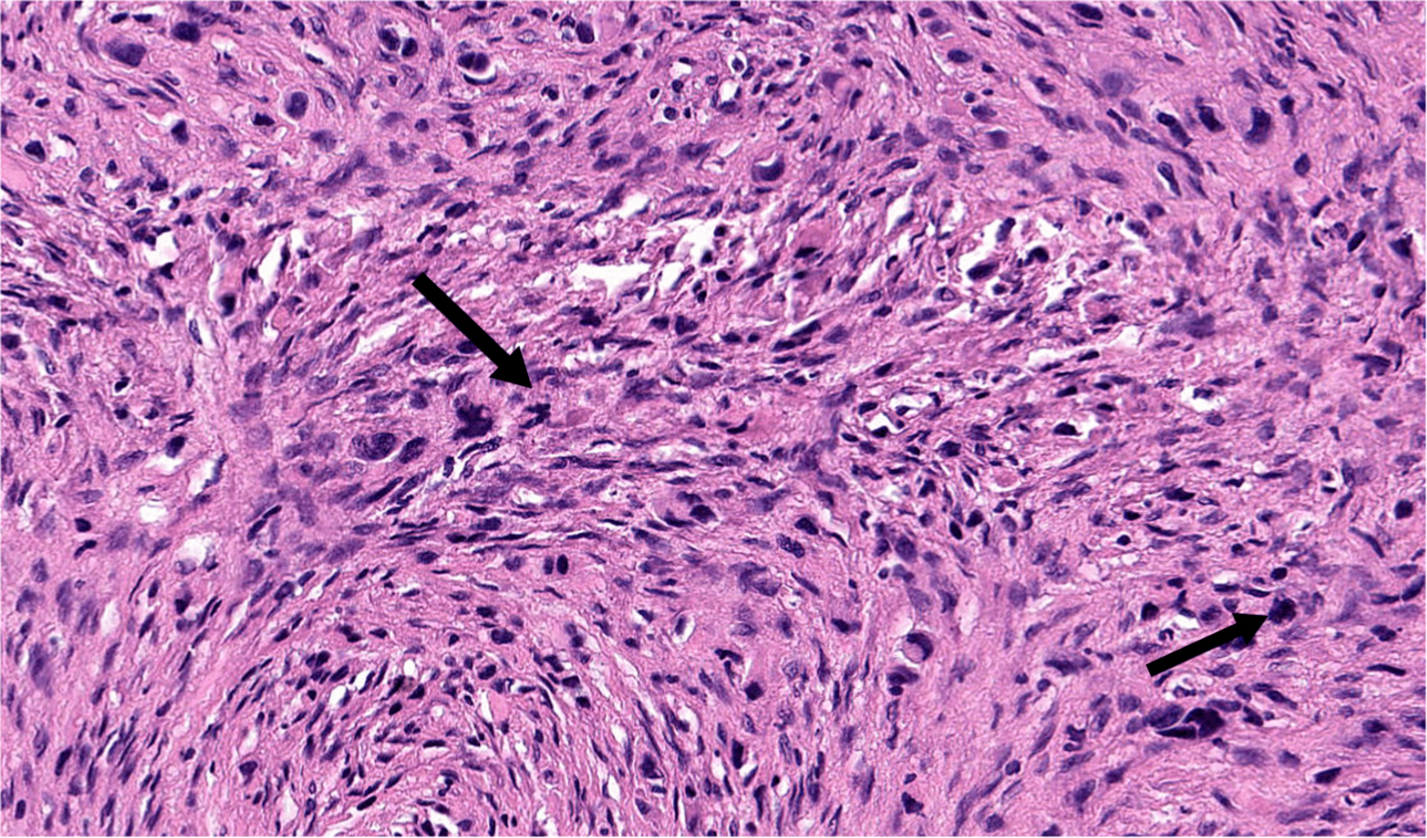
Tumor cells exhibits high grade atypia with brisk mitosis including atypical forms (arrow) - H&E; x400

**Figure 4:**
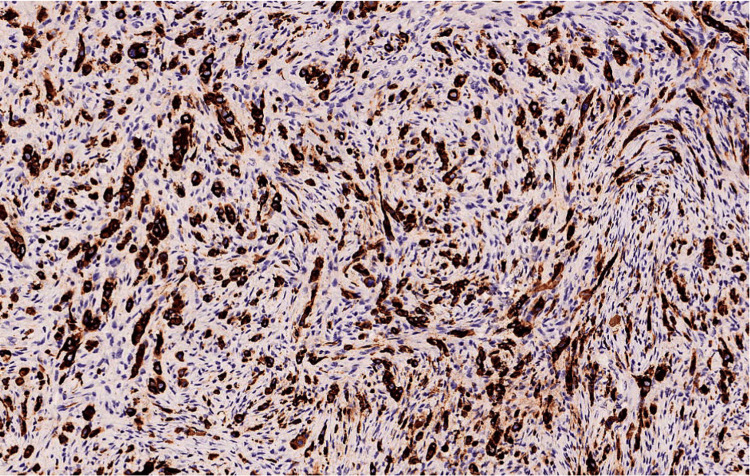
Tumor cells are strongly positive for CK - diaminobenzidine tetrahydrochloride (DAB); x100

**Figure 5:**
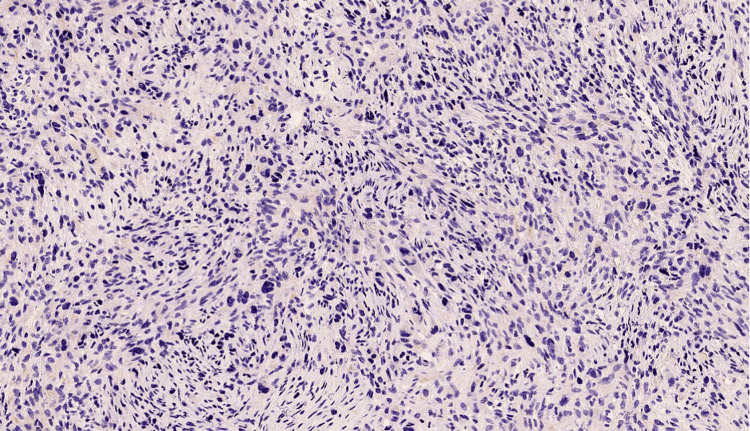
Tumor cells are negative for desmin - diaminobenzidine tetrahydrochloride (DAB); x100

**Figure 6:**
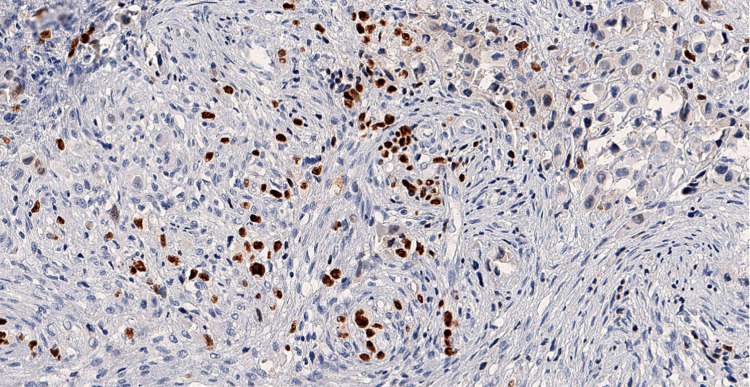
Tumor cells are heterogeneously positive for MyoD1 - diaminobenzidine tetrahydrochloride (DAB); x200

**Figure 7:**
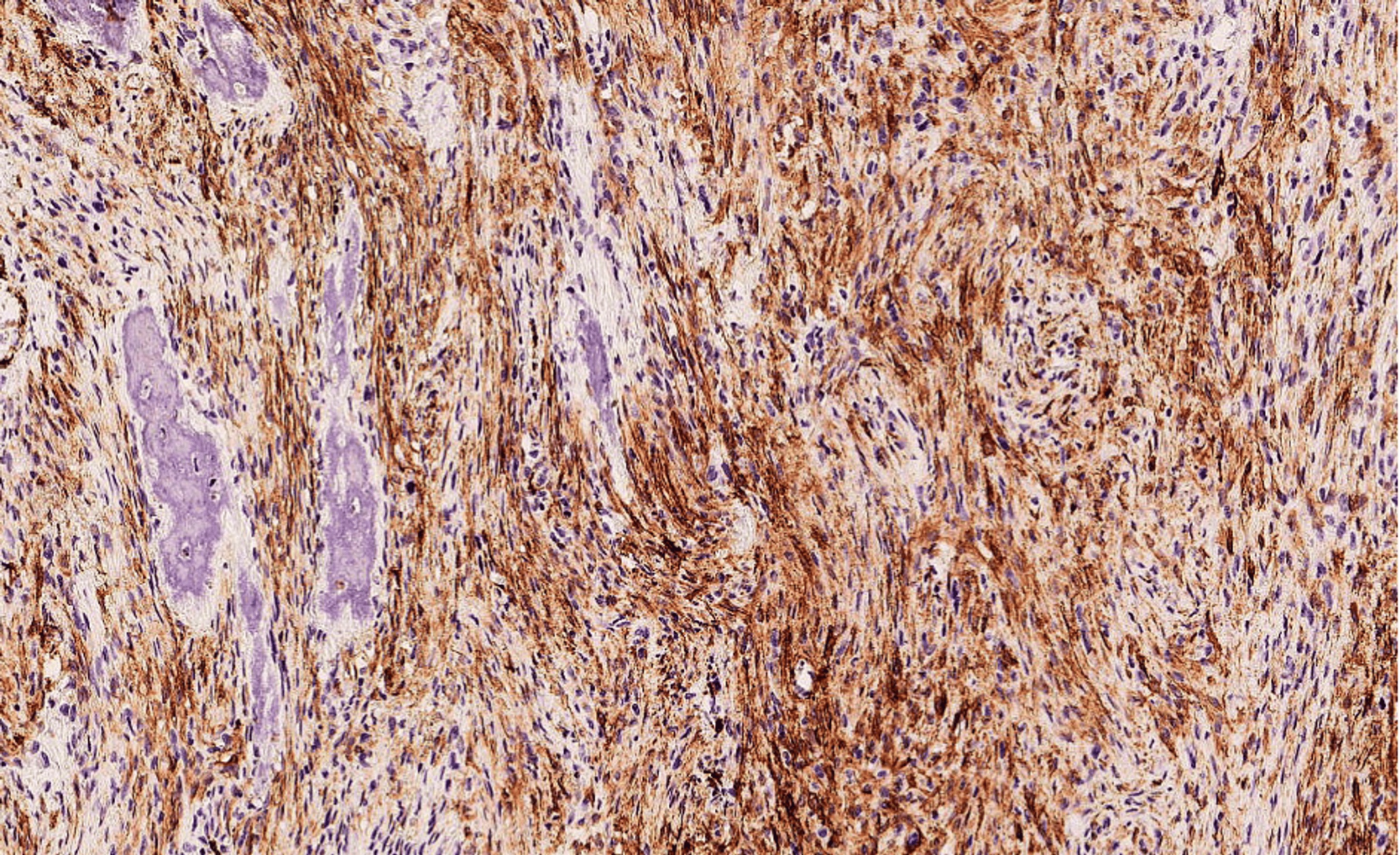
Tumor cells are diffusely positive for smooth muscle actin (SMA) - diaminobenzidine tetrahydrochloride (DAB); x100

**Figure 8:**
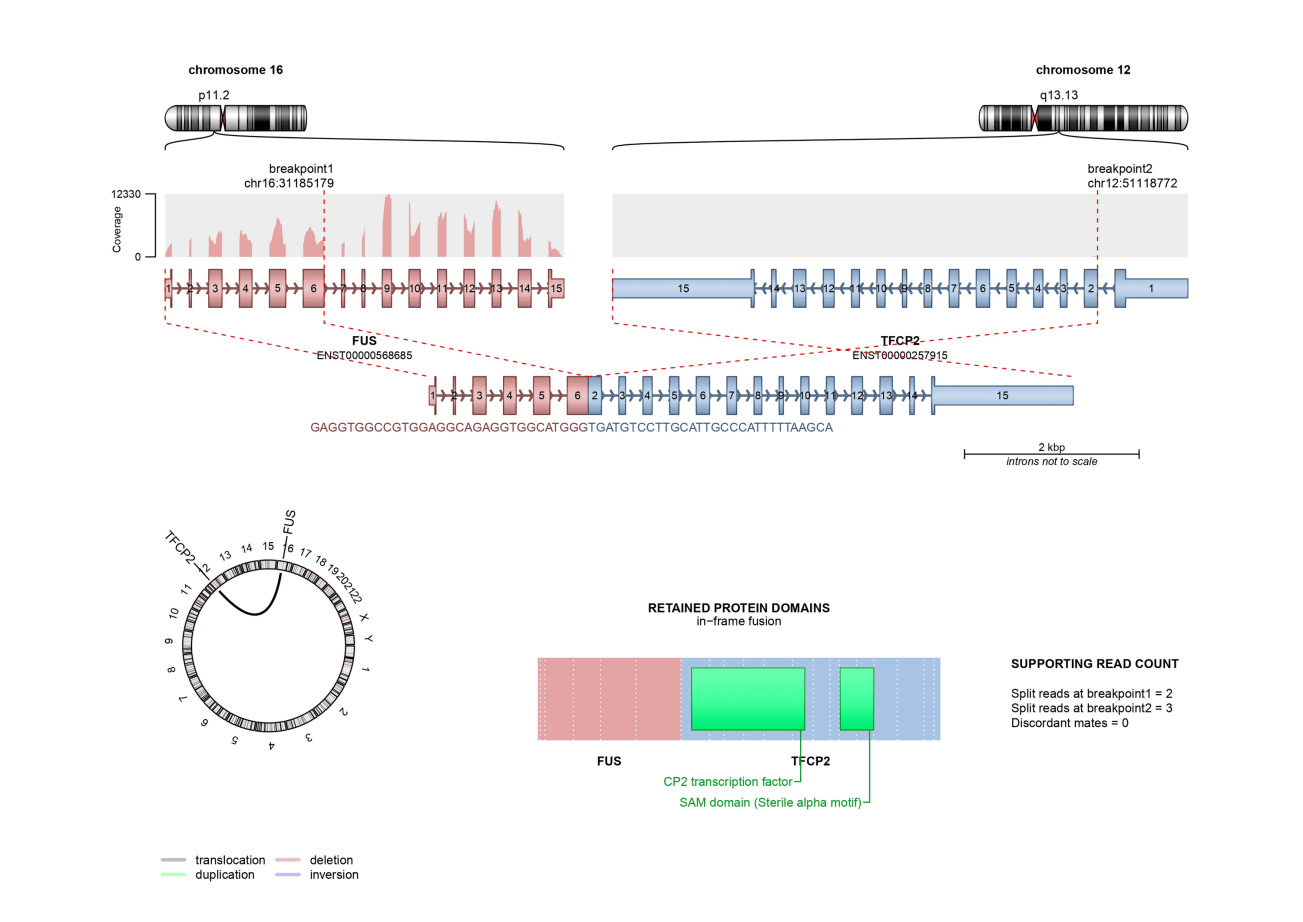
Fusion breakpoint The fusion breakpoint was located at exon 6 of the FUS gene at the 5’ end and exon 2 of the TFCP2 gene at the 3’ end, retaining the complete CP2 transcription and SAM domain. The fusion was predicted to result in an in-frame, translatable transcript, as confirmed by the fusion caller tool Arriba [6]. The fusion breakpoints were visualised using Integrative Genomics viewer. Image provided by MedGenome.

Repeat biopsy (Figures 2, 3) and IHC (Figures 4-7) as detailed in Table 2 were suggestive of sclerosing/spindle cell RMS. Subsequently on NGS sarcoma panel from MedGenome, FUS-TFCP2 fusion positive was detected (Figure 8), suggesting that he was misdiagnosed at presentation.

Contrast-enhanced (CE)-MRI was suggestive of an enhancing lytic lesion in the femoral bone marrow region with associated extra osseous soft tissue component, well-defined mass lesion centered in the right gluteal region largest measuring 17 cm, with metastasis in the pelvic bones and lower end of the right femur.

**Figure 9:**
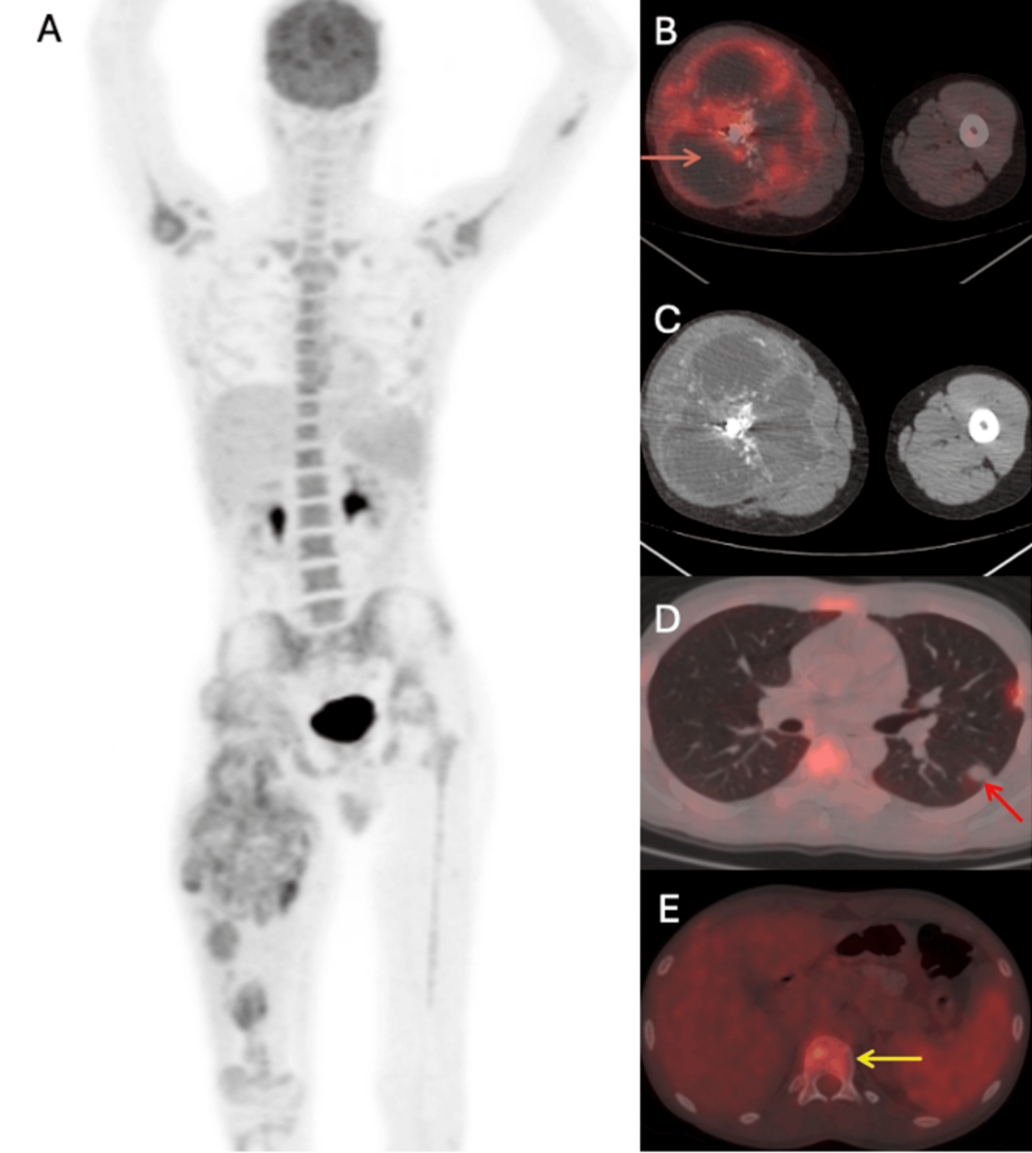
F-18 FDG PET/CT images F-18 fluorodeoxyglucose (FDG) Positron Emission Tomography (PET)/Computed Tomography (CT) images (maximum intensity projection (MIP) –  A, fused axial PET/CT – B, D & E, axial CT – C) for disease status; post intramedullary (IM) fixation of right femur, five cycles of chemotherapy and 10 fractions of radiotherapy, showing heterogeneously enhancing  soft tissue density mass with areas of necrosis and calcifications involving right gluteal and right thigh muscles (orange arrow), faintly FDG-avid peri-fissural left lung lower lobe nodule (red arrow) and multiple FDG-avid lytic-sclerotic lesions involving the skeleton (yellow arrow).

PET-CT (Figure 9) reinforced these findings with standardized uptake value (SUV) max value of 7.8, after which he received four cycles of chemotherapy (VAC). Subsequently he underwent imaging for response assessment, and PET-CT was suggestive of progressive disease. 

Patient received 10 fractions of radiation for pain relief (symptom relief). After which he received one cycle of single agent doxorubicin, and developed disease progression. He then received combination chemotherapy with ifosfamide and etoposide (IE) for three cycles.

**Figure 10:**
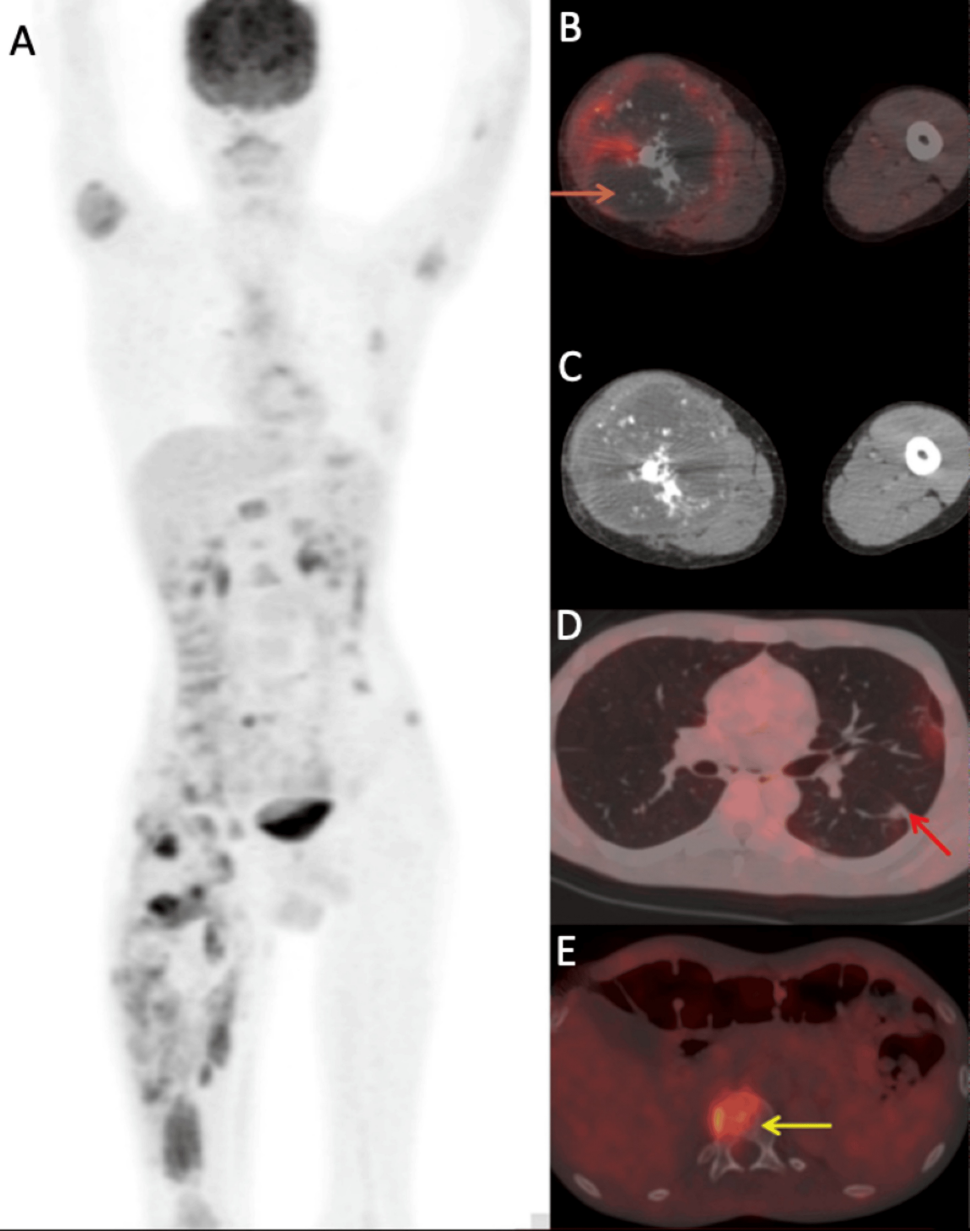
Follow-up F-18 FDG PET-CT images Follow-up F-18 fluorodeoxyglucose (FDG) Positron Emission Tomography (PET)/Computed Tomography (CT) images (maximum intensity projection (MIP) – A, fused axial PET-CT – B, D & E, axial CT – C) for response assessment; on cabozantinib, showing interval reduction in FDG avidity and size of primary mass involving right gluteal and right thigh muscles (orange arrow), peri-fissural left lung lower lobe nodule (red arrow) and skeletal lesions (yellow arrow); suggestive of partial response to therapy.

He progressed on this therapy after three cycles, and was then switched to cabozantinib 40mg OD. Patient is currently on cabozantinib with good clinical response and latest PET-CT scan showing partial response (Figure 10) to therapy with a progression-free survival (PFS) of eight months and follow-up of 24 months since diagnosis.

Case summary 4

**Figure 11:**
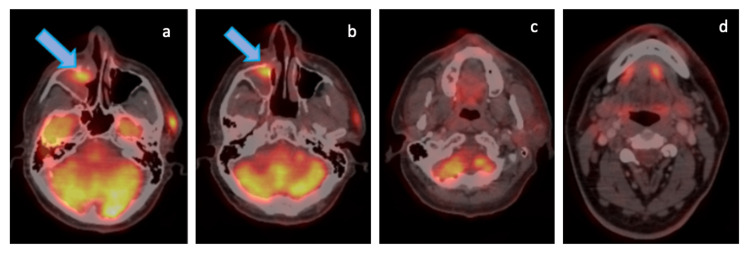
Pretreatment F-18 fluorodeoxyglucose (FDG) Positron Emission Tomography (PET)/Computed Tomography (CT) (a-d)

A 37-year-old male, presented with complaints of watering from right eye and right-side nose and headache. PET-CT scan (Figure 11A-11D) was suggestive of heterogeneously enhancing polypoidal lesion along distal part of right nasolacrimal duct closely abutting right inferior turbinate causing widening of bony nasolacrimal canal with associated bony erosive changes including nasal process of right maxilla. 

Excisional biopsy was suggestive of fibro-osseous neoplasm. However, on IHC, as detailed in Table 2 final report was suggestive of malignant mesenchymal neoplasm (sarcoma), fibroblastic origin.

The patient was referred to our institute and biopsy of right nasal mass was reviewed. Subsequently NGS sarcoma panel from MedGenome revealed EWSR1-TFCP2 fusion oncogenic anomaly.

He was advised surgery for residual tumor, however he denied for the same. Hence, he was started on chemotherapy. After two cycles of chemotherapy (ifosfamide and doxorubicin), he progressed and became metastatic.

**Figure 12:**
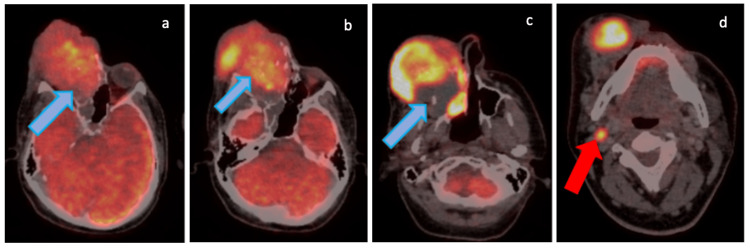
Follow-up F-18 fluorodeoxyglucose (FDG) Positron Emission Tomography (PET)/Computed Tomography (CT) (a-d) demonstrating interval increase in size and metabolic activity of the right nasal mass (solid blue arrows) and appearance of new metastatic right level II cervical lymph node (solid red arrows) suggestive of progressive disease (PD).

**Figure 13:**
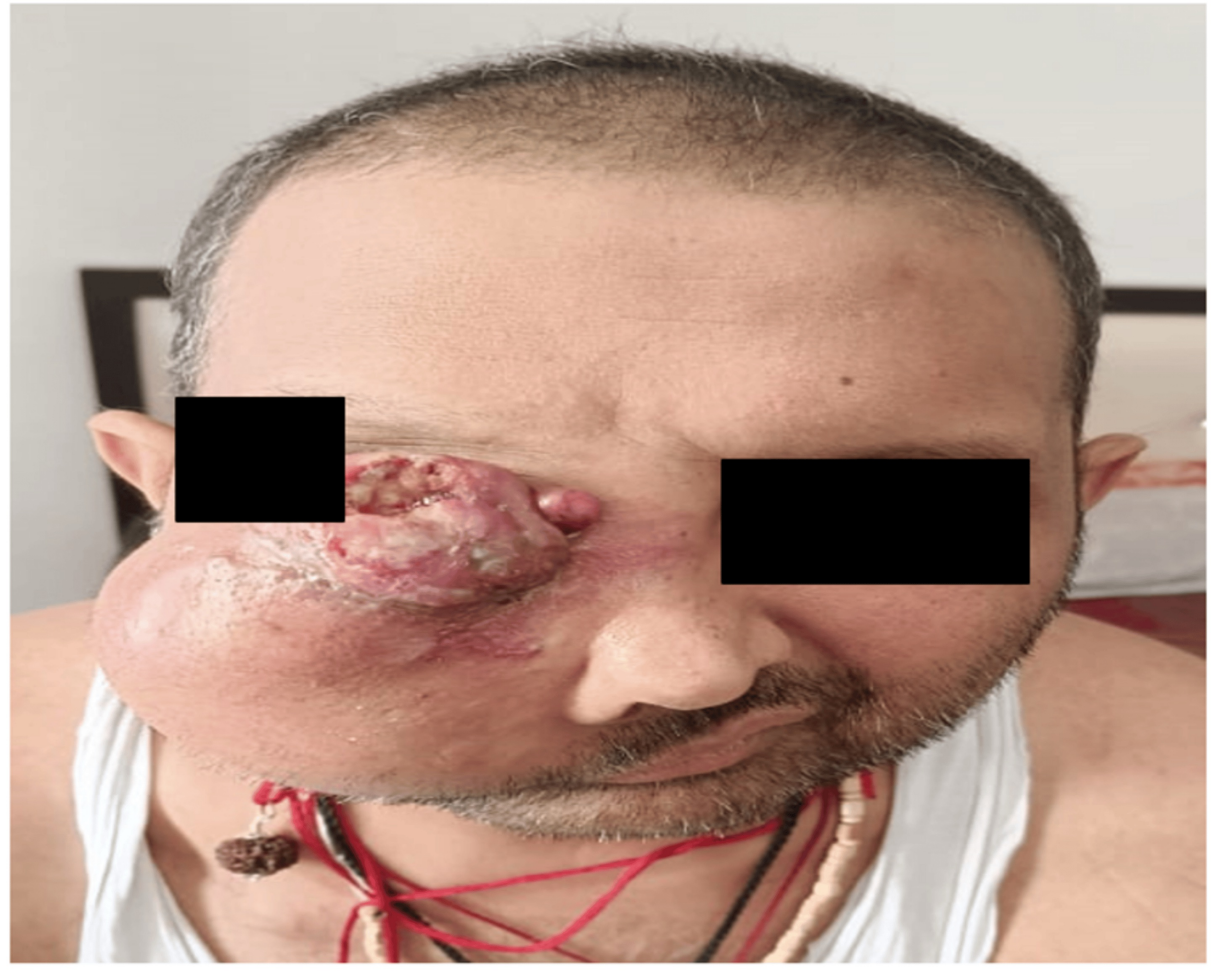
Clinical picture depicting local progression of disease on multiple lines of systemic therapy

**Figure 14:**
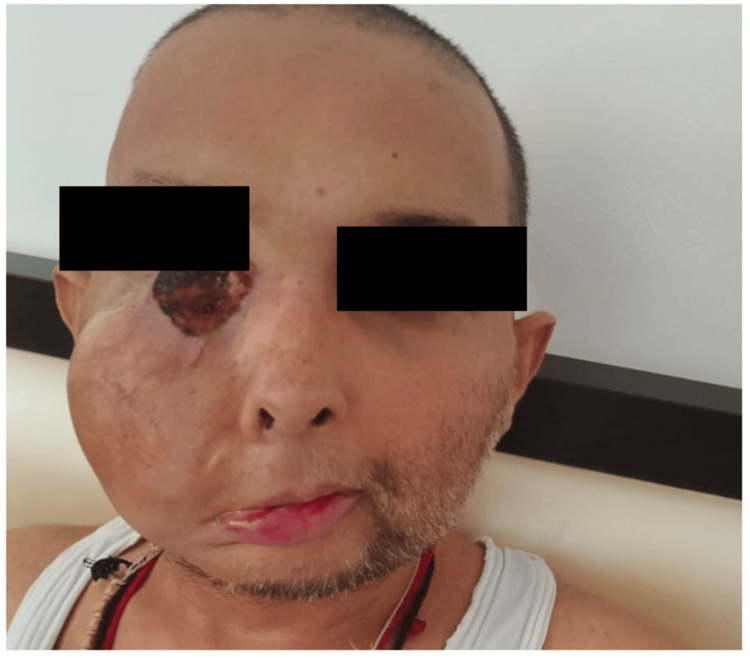
Clinical response after completion of palliative radiation

He was switched to VAC regimen, to which he developed progression (after one cycle) and was switched to IE, followed by two cycles of gemcitabine and docetaxel then one cycle of topotecan, all in view of progression on these regimens. After local progression (Figures 12A-12D, 13) on topotecan his systemic therapy was changed to cabozantinib 40mg OD and he also received palliative radiotherapy to right face (20 Gy in five fractions) (Figure 14 showing post radiation response) followed by continuation of cabozantinib. On recent imaging patient has stable disease, with a PFS of six months and a total follow-up of 17 months since diagnosis.

## Discussion

Intraosseous RMS with TFCP2 gene rearrangement has a marked predilection for young adults with median age of 25 to 30 years [5,7-11]. In our series, the median age was 26.5 years (range- 22-37 years), with all cases having an intra-osseous presentation, similar to those reported in literature. Almost all reported cases of intraosseous RMS with TFCP2 gene rearrangement described in literature occur in head and neck/craniofacial bones. Intraosseous RMS with other alterations, MEIS1-NCOA2 fusion have however been reported in pelvic bones [7]. One of the patients in our series had appendicular skeletal (femur) as primary and was metastatic at presentation in contrast to others who were locally advanced.

TFCP2-mutated tumors express an epithelioid component and show strong and diffuse CK positivity, which may prompt an erroneous diagnosis of sarcomatoid carcinoma, specifically in head and neck tumors with mucosal involvement. In our series, two out of four cases were misdiagnosed and one was not classified, depicting the diagnostic dilemma faced. Moreover, they show negative or downregulated immune-expression of desmin and myogenin, as evident in 50% of cases in our presented series (Table 2). MyoD1 has shown consistent immune-expression in all cases of our series [5,12]. Another peculiarity of this RMS is diffuse immune-expression of ALK. Since there is only a protein expression and no ALK gene rearrangement, there is no cogent evidence of the efficacy of ALK inhibitors in the prevailing literature. Despite that, ALK inhibitors have been tried as a treatment option due to the progressive nature of the disease despite treatment with multimodality approaches.

RMS with TFCP2 fusion has the potential to spread both lymphatically to regional lymph nodes and hematogenously to distant sites, such as the lung and other bones [13-15]. One of our reported cases was metastatic while others were localised at presentation. None of our patients were reported to have any nodal disease. In the metastatic case, site of metastasis differed from what is reported in literature as besides bony metastasis there were soft tissue deposits in the gluteal muscles as well. Another case (Case 4) also became metastatic (lung) while on treatment.

The general treatment paradigm for non-metastatic head and neck RMS (HNRMS) includes maximal safe surgery (often biopsy only for HNRMS) followed by multi-agent chemotherapy and radiotherapy, followed by completion of systemic chemotherapy.

Due to the rarity of RMS with TFCP2 mutations, there are currently no available guidelines for radiation therapy for this specific group of patients. Although, as per the recent 2025 guidelines for RMS, the recommended dose to the primary site in resectable pre- and post-operative tumors is 41.4 Gy in 23 fractions followed by a boost up to 50.4 Gy in 28 fractions for unresectable disease with incomplete response to induction chemotherapy or involved bulky residual lymph nodes [16]. Involved nodal levels receive 41.4 Gy if nodal disease was resected, but elective radiation is usually not done [17]. These guidelines are for RMS in general and not specific to TFCP2-mutated tumors. Also, little is known about the radiosensitivity of this subtype of RMS vis-à-vis other RMS subtypes.

One of our reported cases underwent adjuvant radiotherapy (to tumor bed and preoperatively involved lymph nodes) to a dose of 45 Gy in 25 fractions over five weeks along with two cycles of concurrent chemotherapy with vincristine and cyclophosphamide and two other patients underwent palliative radiotherapy for symptomatic relief, one patient received a dose of 30 Gy in 10 fractions over two weeks to right thigh and another patient to right face to a dose of 20 Gy in five fractions over one week, both of them are currently doing well. The patient receiving radiation over the face responded well with a significant response to radiation. This may point towards the radiosensitivity of these tumors.

Different chemotherapies are used for treatment, such as vincristine, doxorubicin, cyclophosphamide, ifosfamide and etoposide-based regimens. These tumours are chemorefractory to multiple lines of conventional chemotherapies. In our series all four patients received doxorubicin with poor response in two of these patients to single-agent doxorubicin and VAC regimen.

Due to ALK upregulation, ALK inhibitors (crizotinib, alectinib, lorlatinib) have been used with inconclusive results [18,19]. Due to sparsity of data, ALK inhibitors are not usually the preferred option, probably due to extensive prior systemic therapies leading to accelerated tumour evolution, promoting growth of aggressive cell clones, resulting in diminished response to ALK inhibitors [20]. To fully evaluate their clinical potential, ALK inhibitors can probably be tried in therapy-naive patients. In our series, one patient receiving cabozantinib had good response and one had stable disease post progression on multiple other chemotherapy regimens, pointing towards good clinical activity of tyrosine kinase inhibitors (TKIs).

Regular monitoring of these patients is strongly recommended as they have poor outcomes and tend to recur locally as well, and have a high propensity of metastasis [10,12]. Current literature shows a 20% risk of regional lymph node metastasis and a 50% risk of developing distant metastasis with a 35% disease-free survival (DFS) at two years. Also, due to paucity of data available regarding the long-term outcomes, follow-up for longer periods would be advisable.

A diagnosis of craniofacial tumor in young males with biopsy suggesting an intraosseous sarcoma should point towards testing for TFCP2 mutation. In our opinion, aggressive multimodality treatment should be offered to this set of patients as they have an aggressive course with high risk of local and regional recurrence as well as systemic dissemination. In light of all available literature, this being an uncommon disease, it should entail multidisciplinary tumor board discussion with informed decision-making with the patient.

## Conclusions

Intra-osseous RMS with TFCP2 mutation occurs predominantly in head and neck region, in young adults and is a distinct entity with diagnostic and therapeutic challenges, while the prognosis remains guarded. It is refractory to conventional chemotherapy but seems to be radiosensitive, which should be further evaluated in larger cohorts. This is the first Indian case series of its kind although with a limited sample size, and has shown a clinical benefit of combining radiation (even in palliative settings) and TKIs for patients with TFCP2 mutations. This case series could be a harbinger for further larger series from India and establishing a multi-institutional registry could help in collating data for such patients. Due to lack of data and treatment protocols in place, a timely and correct diagnosis combined with individualization of multimodality treatment and its sequencing can help in reducing the morbidity of treatment and may also help in improving outcomes.
